# Altered Spontaneous Brain Activity in Betel Quid Dependence Chewers: A Resting-State Functional MRI Study With Percent Amplitude of Fluctuation

**DOI:** 10.3389/fpsyt.2022.830541

**Published:** 2022-05-02

**Authors:** Lili Fu, Huijuan Chen, Tao Liu, Liting Liu, Qingqing Fu, Weiyuan Huang, Feng Chen

**Affiliations:** ^1^Department of Radiology, Hainan General Hospital (The Hainan Affiliated Hospital, Hengyang Medical School, University of South China), Haikou, China; ^2^Department of Radiology, Hainan General Hospital (Hainan Affiliated Hospital of Hainan Medical University), Haikou, China; ^3^Department of Neurology, Hainan General Hospital (Hainan Affiliated Hospital of Hainan Medical University), Haikou, China

**Keywords:** betel quid, betel quid dependent, drug dependence, resting-state fMRI, percent amplitude of fluctuation

## Abstract

**Objective:**

This study aimed to investigate brain spontaneous neural activity changes in betel quid dependence (BQD) chewers using the percent amplitude of fluctuation (PerAF) method.

**Methods:**

This study included 48 BQD chewers. The healthy control (HC) group comprised 35 volunteers who were matched with BQD chewers in age, gender, and educational status. All subjects underwent resting-state functional magnetic resonance imaging (rs-fMRI) and neuropsychological tests. The PerAF method was used to identify BQD-related regional brain activity changes. An independent samples *t*-test was used to evaluate the PerAF difference across two groups. The association between PerAF changes and clinical features such as BQD scores, duration of BQD, Hamilton Depression Rating Scale-24 item (HAMD-24), and Hamilton Anxiety Rating Scale-14 item (HAMA-14) was evaluated by using Spearman's correlation analysis. It assessed the ability of the PerAF method to distinguish between BQD chewers and HCs using a receiver operating characteristic (ROC) curve.

**Results:**

Compared to the control group, BQD chewers showed decreased PerAF in right anterior cingulate cortex (ACC), right middle frontal gyrus (MFG), right insula, right precuneus, left putamen, left supramarginal gyrus (SMG), and left cerebellum and increased PerAF in right orbitofrontal and left superior temporal gyrus (STG) [*P* < 0.05, Gaussian random field (GRF) corrected]. PerAF values of the right MFG and right ACC had a significant negative relationship with the duration of BQD (*P* < 0.05). The average values of PerAF in the left putamen, left cerebellum, and left STG showed significant discriminatory power in distinguishing BQD chewers from HCs, with relatively prime area under the curve (AUC) values.

**Conclusion:**

Our findings suggested that betel quid chewing is associated with spontaneous neural activity alterations in the impulsivity areas (MFG and ACC), cognitive (MFG, ACC, precuneus, and the cerebellum), and reward (orbitofrontal, putamen, and insula) systems, which may be correlated with neuropathological mechanisms of BQD. Also, PerAF may be useful as a potential sensitive biomarker for identifying spontaneous brain activity changes in BQD chewers.

## Introduction

Betel quid (BQ) is a psychoactive stimulant that, after ethanol, nicotine, and caffeine, is the most commonly used addictive substance (1). There are over 600 million BQ consumers globally, most of whom they concentrated in Asia and Pacific islands (2). The term “betel quid” refers to a mixture of ingredients that includes areca nut (AN), piper betel leaf (a common vine), and slaked lime (calcium hydroxide), but the composition of quid varies among different populations and areas (3, 4). The parasympathomimetic characteristics of BQ stimulate nicotine and muscarinic receptors. Therefore, chewing BQ usually leads to a reliance syndrome, which is featured with increased attention, mild pleasure, comfort, and postprandial satisfaction, as well as a withdrawal syndrome that includes insomnia, mood swings, anxiety, and irritability (5). BQ chewing is a public health concern since that can lead to a range of health problems, such as oral cavity cancer and many precancerous lesions associated with leukoplakia and submucosal fibrosis of the oral cavity (6). The International Agency for Research on Cancer has classified BQ as a human carcinogen (3, 7). However, at present, the mechanism behind betel quid dependence (BQD) has remained unclear. Most studies have been restricted to either epidemiological or biological studies (8, 9). Only a few studies have been conducted to determine the behavioral and psychological characteristics associated with individuals starting and/or maintaining BQ use.

Due to the advantages of being non-invasive, easy to get signals, pretty high space-time resolution, and requirement of a minimum workload from patients, the resting-state functional magnetic resonance imaging (rs-fMRI) has been widely used to reveal abnormal spontaneous brain activity. The amplitude of low-frequency fluctuation (ALFF), fractional ALFF (fALFF), and Regional Homogeneity (ReHo) are the three common parameters to address regional brain alterations (10–16). However, due to the arbitrary unit of blood oxygenation level-dependent (BOLD) signals, ALFF cannot be immediately applied to further statistical analysis at the group level. Besides that, ReHo cannot reflect accurately the activity of neurons in a specific integrin because it is based on time consistency.

A new method, the percent amplitude of fluctuation (PerAF), has been proposed, which is based on the percentage of signal alterations in the task functional magnetic resonance imaging (fMRI) (17). Even though there is no explicit task vs. control design in rs-fMRI, an index similar to the percentage of signal alterations can be developed by calculating the percentage of BOLD fluctuations compared to mean BOLD signal changes for every point in time, then measuring the mean value across the entire time sequence, that is namely the “percent amplitude of fluctuation” or PerAF (18). To further standardize the data, the PerAF of each voxel can be divided by the globally averaged PerAF of each individual, resulting in mPerAF. When compared to ALFF, PerAF and mPerAF can perform group-level data analysis directly. In fractional ALFF, PerAF can also eliminate the perplexing combination of voxel-specific fluctuation amplitude (18). PerAF has been proven in several studies to be more reliable than ALFF, ReHo, and degree centrality (17, 19). Therefore, PerAF is a relatively reliable, effective, and direct index for voxel-level-based rs-fMRI research and thus a promising index. The increase of PerAF means the enhancement of spontaneous neural activity, while the decrease of PerAF means the diminution of that.

Previous studies have linked BQ chewing and dependence to spontaneous brain activities. BQD patients had significantly slowed ALFF and ReHo values in the prefrontal gyrus and left fusiform and significantly higher ALFF and ReHo values in the primary motor cortex area, temporal lobe, and some occipital lobe regions, which could reflect the neural plasticity of the cerebral functional network caused by BQD (20). A voxel-based analysis had revealed that BQD chewers had a higher mean fractional amplitude of low-frequency fluctuation (mfALFF) activation of the left cuneus and precuneus than the healthy control (HC) (21). Despite this, no studies have used PerAF to investigate spontaneous changes in brain activity in BQD chewers. We hypothesized that BQD chewers had altered spontaneous brain activity in this investigation, and the PerAF method could detect these alterations. To verify this hypothesis, PerAF was used to detect global spontaneous neural activity in BQD chewers, which may give insight into the neurobiological mechanisms underlying BQD.

## Methods

### Ethics Statement

The Research Ethics Review Committee of the Hainan General Hospital, based on the Declaration of Helsinki (2000), formally approved this study. Written informed consent was obtained from each participant before starting the research.

### Inclusion and Exclusion Criteria

All participants were Hainanese natives. The following were the BQD chewer's inclusion criteria: (1) the BQD subjects should be between the ages of 18 and 60; (2) the BQD subjects should not use nicotine or have used nicotine only one time or two times a month over the previous 3 years. The Fagerstrom Test for Nicotine Dependence (FTND) was also used to test nicotine addiction status to avoid nicotine's influence; (3) BQD subjects with Betel Quid Dependence Scale (BQDS) > 4, Hamilton Depression Rating Scale-24 item (HAMD-24) ≤ 7, and Hamilton Anxiety Rating Scale-14 item (HAMA-14) ≤ 7; (4) no contraindication to magnetic resonance imaging (MRI) examination; no structural lesions and abnormal signals in craniocerebral MRI, and the imaging data was complete; (5) not addicted to any other substances and not taking any antidepressants or other psychotropic or addictive drugs; (6) no systemic illnesses and familial psychiatric history; and (7) right-handed.

The following were the inclusion criteria for the HC participant: (1) age range of 18–60 years; (2) no use of BQ, AN, and cigarette in any form; (3) no MRI contraindications; (4) no structural lesions or abnormal signals in craniocerebral MRI, and the imaging data were complete; (5) no additional substance abuse or addiction, and no antidepressants or other psychotropic or addictive drugs; (6) no systemic illnesses or psychiatric history in the family; and (7) right-handed. Finally, 48 BQD chewers and 35 HC volunteers were recruited from Hainan province's local community.

### Questionnaire

We evaluated all subjects with a questionnaire including age, gender, educational status, the daily dosage of BQ, duration of BQD, and the usage of tobacco and alcohol before MRI examination. The BQDS was used to assess BQD. This scale quantitatively defines the degree of dependence of BQD individuals, and it has been tested to have good internal consistency and validity. Thus, BQDS is currently the most widely used BQD evaluation tool (22, 23). On the scanning day, the HAMD-24 and HAMA-14 scales were used to assess depression and anxiety levels.

### MRI Data Acquisition

The rs-fMRI data were acquired using a 3-T MRI scanner with a standard 32-channel head coil (TIM Skyra, Siemens Medical Solutions, Erlangen, Germany). During the MRI scanning, all subjects were told to keep their heads motionless, keep their eyes open while thinking of nothing, and use foam paddings to reduce head movement. A gradient-echo echo-planar imaging (GRE-EPI) sequence (repetition time (TR) = 2,000 ms, echo time (TE) = 30 ms, field of view (FOV) = 224 × 224 mm^2^, image matrix = 64 × 64, section thickness = 3.5 mm, each brain volume consists of 32 axial slices, each functional run contains 240 brain volumes) was used to obtain the BOLD of whole-brain functional images. A magnetization-prepared rapid gradient-echo (MPRAGE) sequence was used to obtain the high-resolution T1-weighted structural image (TR = 2,530 ms, TE = 2.98 ms, FOV = 256 × 256 mm^2^, in-plane matrix = 256 × 256, 192 sagittal slices with a thickness of 1 mm). Then, a routine MRI scanning was performed to rule out any gross cerebral pathology.

### Data Preprocessing

The preprocessing of fMRI imaging data was performed using the REST plus V1.2 (http://www.restfmri.net) toolbox, which included: (1) removing the first ten functional volumes; (2) slice timing correction; (3) head motion correction; (4) co-registration, spatial normalization, and resampling to 3 × 3 × 3 mm^3^; (5) smoothing the resampled images with an isotropic Gaussian kernel of 6 mm; (6) removal of linear trends; (7) covariate regression with the Friston-24 parameter model (24, 25) to remove the nuisance signals; and (8) band-pass (0.01–0.08 Hz) filtering to remove the influence of high-frequency noise and low-frequency drift. Participants with head motion more than 1.5 mm of maximal translation or 1.5° of maximal rotation were excluded from further analyses. No participants were excluded due to excessive head motion in this research. PerAF was calculated after data preprocessing. PerAF, mPerAF, and zPerAF maps were then generated.

### Statistical Analysis

SPSS version 25.0 (IBM Corp, Armonk, NY, USA) was used to compare the demographic and clinical features of BQD chewers and HC volunteers. The normality test was performed before the comparison analysis for continuous variables. An independent two-sample *t*-test was adopted if the variables met the normal distribution criteria. If not, an independent two-sample nonparametric test was used to analyze the data. An independent two-sample *t*-test was adopted for continuous variables (age, HAMA-14, and HAMD-24), a chi-square test was performed for gender, and an independent two-sample nonparametric test was used to analyze years of education. *P* < 0.05 was considered to be statistically significant. The data were shown as mean ± standard deviation (mean ± SD). Two-sample *t*-tests were performed to explore the intergroup differences in PerAF between BQD and HC using the DPABI software. To eliminate potential confounding factors, age, gender, and educational status were regressed out (26). Multiple comparisons were conducted with the Gaussian random field (GRF) correction, voxel-wise *P* < 0.01, and cluster-level *P* < 0.001. Regions of interest (ROIs) were identified as brain regions with statistical PerAF alterations between BQD chewers and HC. We extracted the PerAF values from the ROIs. With a significant level of *P* < 0.05, Spearman's correlation analysis was performed between the PerAF values of ROIs and BQDS, dosage, duration, HAMA-14, and HAMD-24. Age, gender, and educational status were all regressed. A receiver operating characteristic (ROC) curve analysis was conducted to assess the PerAF method's ability to distinguish between BQD chewers and HCs.

## Results

### Demographics and Clinical Characteristics

Eighty-three participants (48 BQD chewers and 35 control volunteers) were included in the ultimate statistical analysis. The mean age of BQD chewers was 37.9 ± 10.9 years old, and the mean education was 12.1 ± 2.7 years. The mean age of HC volunteers was 41.9 ± 11.5 years old, and the mean education was 12.9 ± 2.6 years. BQD chewers revealed that they had been chewing BQ for an average of 13.3 ± 8.5 years, with a range of 4.8–21.8 years. The mean BQDS score in the BQD group was 8.6 ± 3.0. HAMA-14 and HAMD-24 did not meet clinically meaningful thresholds on average. The demographics and clinical characteristics of BQD and HC subjects were demonstrated in Table 1. There was no statistical difference between the BQD chewers and HCs in terms of age, gender, education level, HAMA-14, or HAMD-24 (all *P* > 0.05).

**Table 1:** Demographics and clinical characteristics of participants.

	**BQD (*n* = 48)**	**HC (*n* = 35)**	***P*** **-value**
Gender (males / females)	36 / 12	24 / 11	0.518^a^
Age (year)	37.9 ± 10.9	41.9 ± 11.5	0.110^b^
Education (year)	12.9–15	12.12–15	0.180^c^
BQDS(score)	8.6 ± 3.0	N / A	
BQ dosage (g / d)	70.0 ± 63.2	N / A	
Duration of BQD (year)	13.3 ± 8.5	N / A	
HAMA-14 (score)	1.6 ± 1.8	2.3 ± 1.8	0.075^b^
HAMD-24 (score)	2.0 ± 2.2	2.6 ± 2.5	0.255^b^

*Values are expressed as means ± standard deviations except for gender and education, gender is presented as a number, education is expressed as median and inter-quartile range. BQ, betel quid; BQD, betel quid dependence; BQDS, betel quid dependence scale; HAMA-14, hamilton anxiety rating scale-14 item; HAMD-24, hamilton depression rating scale-24 item; HC, healthy control; N / A, not applicable*.

a *P-value between the two groups was obtained by chi-square test*.

b *P-value between the two groups was obtained by an independent sample t-test*.

c *P-value between the two groups was obtained by independent sample non-parametric test*.

### Group Differences in PerAF

**Figure 1:**
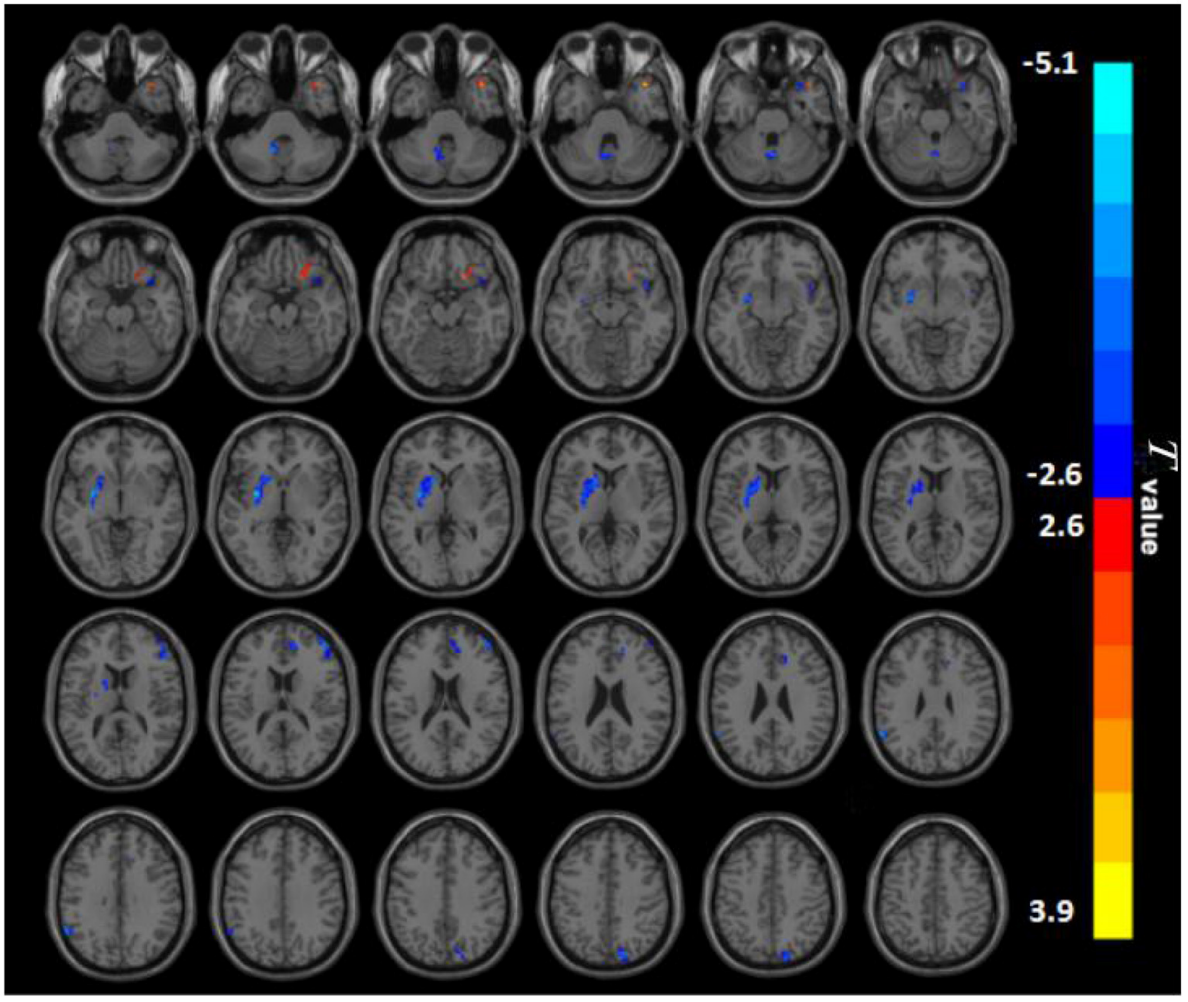
PerAF maps show differences between BQD individuals and HC subjects (*P* < 0.05). The BQD group showed significantly lower PerAF values in the right anterior cingulate cortex, right middle frontal gyrus, right insula, right precuneus, left putamen, left supramarginal gyrus, left cerebellum, but increased PerAF values in the right orbitofrontal, left superiortemporal gyrus relative to both the HC group. BQD, betel quid dependence; HC, healthy control; PerAF, percent amplitude of fluctuation. The color bar represents *T*-values for the two-sample *t*-test. Colors in red and blue indicate significant increase and decrease in the two-sample *t*-test respectively.

Between the two groups, there were significantly distinct patterns of spontaneous neural activity. Compared with HC, BQD chewers exhibited decreased PerAF values in the right anterior cingulate cortex (ACC), right middle frontal gyrus (MFG), right insula, right precuneus, left putamen, left supramarginal gyrus (SMG), and left cerebellum, while that exhibited increased PerAF values in the right orbitofrontal and left superior temporal gyrus (STG) (Figure 1; Table 2).

**Table 2:** Brain regions showing differences in PerAF between BQD and HC groups.

**Brain region**	**Voxel**	**Peak *T* score**	**MNI coordinates**		
			**X**	**Y**	**Z**
**BQD < HC**					
R.ACC	26	−3.3138	15	45	18
R.MFG	38	−3.9018	45	48	18
R.Insula	32	−3.5913	30	15	−27
R.Precuneus	27	−3.5871	15	−81	42
L.Putamen	207	−5.0539	−30	−3	0
L.SMG	22	−4.451	−60	−51	30
L.Cerebellum	42	−4.2455	−12	−54	−36
**BQD > HC**					
R.Orbitofrontal	28	3.2213	21	21	−15
L.STG	20	3.9199	39	18	−30

*ACC, anterior cingulate cortex; BQD, betel quid dependence; HC, healthy control; L, left; MFG, middle frontal gyrus; PerAF, percent amplitude of fluctuation; R, right; SMG, supramarginal gyrus; STG, superiortemporal gyrus*.

*T, statistical value of peak voxel showing PerAF values differences between the two groups (negative values: BQD < HCs; positive values: BQD > HCs); MNI, montreal neurological institute coordinate system or template; x, y, z, coordinates of primary peak locations in the MNI space*.

### Correlation Analysis

**Figure 2:**
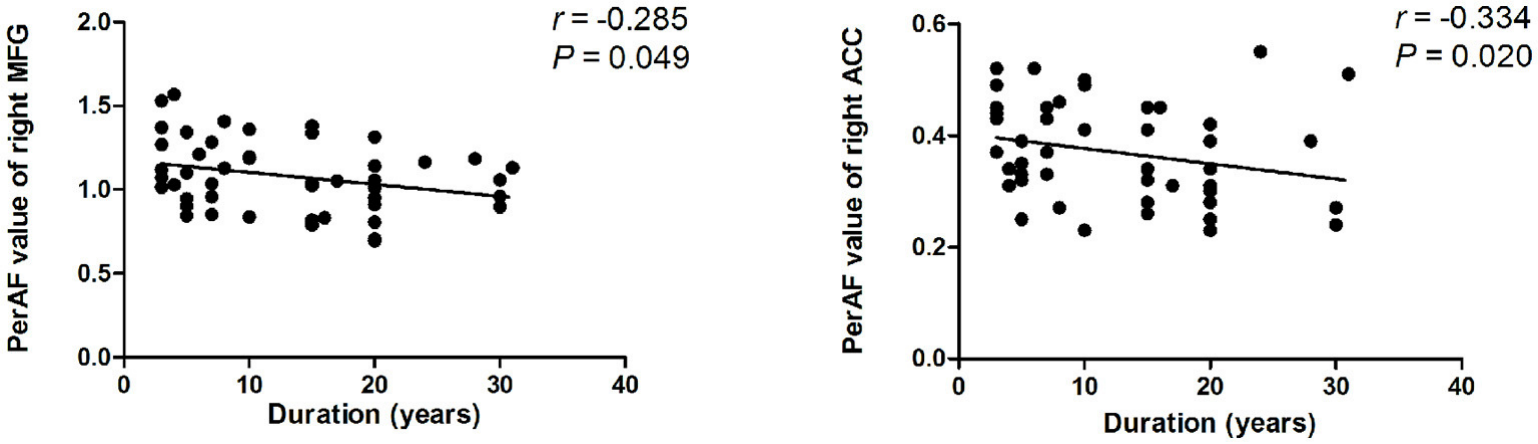
Correlation results between PerAF value and duration of BQD. Spearman correlation analyses reveal that PerAF value of the right MFG, the right ACC showed a negative correlation with duration of BQD in BQD individuals (*P* < 0.05). ACC, anterior cingulate cortex; BQD, betel quid dependence; MFG, middle frontal gyrus; PerAF, percent amplitude of fluctuation.

There is a negative association between PerAF values of the right MFG, the right ACC, and the duration of BQD (*r* = −0.285, *P* = 0.049; *r* = −0.334, *P* = 0.020; Figure 2). No correlation was found between BQDS and spontaneous neural activity alterations. There is also no link found between HAMA-14, HAMD-24, and neural activity alterations, suggesting that anxiety and depressive mood in the BQD group might not be the contributors to the decreased activity of these brain areas.

### ROC Curve

**Figure 3:**
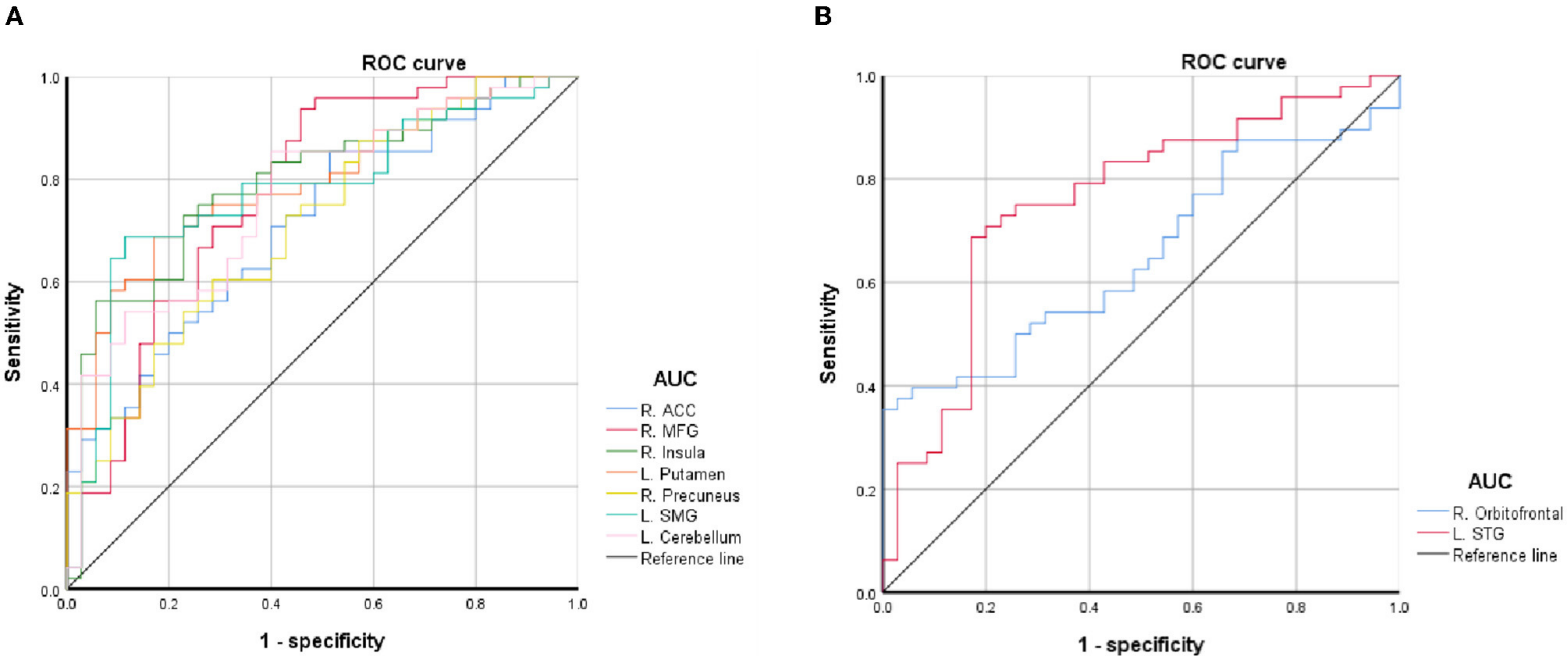
ROC curve of PerAF differences in brain areas. **(A)** Decreased PerAF areas; **(B)** Increased PerAF areas. ACC, anterior cingulate cortex; AUC, area under the curve; L, left; MFG, middle frontal gyrus; PerAF, percent amplitude of fluctuation; R, right; ROC, receiver operating characteristic; SMG, supramarginal gyrus; STG, superiortemporal gyrus.

The average values of PerAF in the brain regions with spontaneous neural activity changes were extracted for ROC curve analysis. Our data indicated that these BQD-related regional brain activity changes exhibited differences between the BQD chewers and HCs. They might serve as potential sensitive biomarkers for identifying the two groups (Figure 3; Table 3).

**Table 3:** ROC curve for PerAF differences in brain regions between BQD and HC groups.

**Brain region**	**Area under the curve**	**Sensitivity**	**Specificity**	**Cut off points^a^**
R.ACC	0.714	85%	49%	0.3701
R.MFG	0.773	94%	54%	1.1879
R.Insula	0.793	56%	43%	0.6892
R.Precuneus	0.713	60%	71%	1.0827
L.Putamen	0.792	69%	83%	0.5490
L.SMG	0.775	69%	89%	0.5740
L.Cerebellum	0.764	85%	60%	0.6910
R.Orbitofrontal	0.652	35%	100%	1.1335
L.STG	0.758	69%	83%	0.8504

*ACC, anterior cingulate cortex; BQD, betel quid dependence; HC, healthy control; L, left; MFG, middle frontal gyrus; PerAF, percent amplitude of fluctuation; R, right; ROC, receiver operating characteristic; SMG, supramarginal gyrus*.

*STG, superiortemporal gyrus*.

a *cut off point of mean PerAF signal value*.

## Discussion

This study is the first to identify BQD-induced brain alterations using PerAF. Our results revealed abnormal PerAF in some impulsivity areas (MFG and ACC), cognitive areas (MFG, ACC, precuneus, and the cerebellum), and reward systems such as orbitofrontal, putamen, and insula in the brain in BQD chewers. Our results suggest that PerAF is an effective tool for investigating spontaneous brain activity alterations in individuals with BQD. Moreover, ROC curve analysis revealed the left putamen, left cerebellum, and left STG had a relatively high area under the curve (AUC) values, indicating that these BQD-related regional brain activity changes might serve as potential sensitive biomarkers for identifying the two groups.

A decreased PerAF value of the right MFG was observed in the BQD group. The MFG, which is encompassed in the dorsolateral prefrontal cortex (dlPFC), is well-known for its role in impulse control and executive functions (27). Dysfunctions in dlPFC have been linked with drug addiction, which leads to compulsive drug taking, yearning, denial of disease, and a loss of enthusiasm to seek medical assistance (28). The cortical thickness of the dlPFC was found to have a significant role in mediating executive function deficits among BQD chewers in a recent study (29). Moreover, dysfunction of functional activity in the dlPFC may relate to diminished cue-induced hankering and responding suppression in BQD (30). The dlPFC is also involved in decision-making (31, 32). A previous fMRI study demonstrated that reduced neural activity in the prefrontal gyrus within methamphetamine-dependent participants was related to maladaptive decision-making (33). Furthermore, dlPFC has to be well known for its contribution to resolving conflicts. For example, in people who had restarted cigarette consumption, dlPFC was associated with enhanced cognitive control and a greater ability to resolve conflicts (34). According to previous studies and observation of this study, we speculated that the decreased PerAF value in the right MFG within the dlPFC may contribute to demonstrating the affects of BQD on cerebrum impulsivity and the cognitive system.

Similar to MFG, ACC is also contained in the impulsive and cognitive systems. According to the prominent hypothesis of control conflict (35), ACC symbolizes the emergence of response conflict, which gives rise to the agglomeration of dlPFC for better cognitive management of subsequent behavior. Moreover, various neuroimaging studies have shown that cognitive management is associated with a special cortical-subcortical network, including ACC and dlPFC (36). Our previous research indicated decreased ALFF and ReHo values in the right rostral ACC in BQD individuals (20). When compared to HCs, BQD chewers had lower functional connectivity (FC) from ACC to their default mode network (DMN) (37). As far as we know, ACC is also reported to be responsible for the inhibitory control of reward-related behavior (38). The decreased PerAF value in the right ACC and right MFG within the dlPFC in this study may be associated with compulsive BQ chewing behavior, cognitive management, decision-making, and leading to a certain degree of craving.

The duration of BQD was negatively correlated with decreased PerAF of right MFG and right ACC, which demonstrated that the longer a person chewed BQ, the lower their PerAF of MFG and ACC became. Therefore, we deduced that BQD duration might be associated with altered brain function. Given the importance of the frontal cortex and limbic system in the addiction process, it seems sensible to associate functional changes with the duration of BQD. Yet, the correlation result needs further confirmation. The correlation was only found between some brain areas and the duration of BQD, and no correlation was found in BQDS, suggesting that the BQD duration may have a greater impact on brain function or that it may be due to the relatively low BQD score (BQDS, 8.6 ± 3.0). The small sample size might have accounted for the results.

Besides, lower spontaneous brain activity was observed in the BQD group in the precuneus, which is part of the brain areas of the DMN. The DMN, contained in the cognitive system, is involved in a variety of brain functions which include auditory attention, visuality, memory, language processing, and motor performance (39, 40). As a key component of the DMN, the precuneus is closely associated with determining visual and appetite cues (41, 42), which means that it is involved in visuospatial processing (43). As a result, we contend that the decreased PerAF of the precuneus in BQD chewers may be related to appetite cues and visuospatial processing. Similar to our findings, a recent structural imaging study found a significant decrease in cortical thickness in the precuneus of BQD individuals, which could be related to neurodegeneration caused by chronic BQ chewing (44).

In addition to the alterations in cognitive areas, altered brain activity was found in reward system-associated areas such as orbitofrontal, putamen, and insula in BQD chewers. Increased PerAF was found in the orbitofrontal cortex (OFC) in individuals with BQD, which forms part of the prefrontal cortex (PFC). It is reported that the orbitofrontal network enhances the capacity to control behavior based on potential consequences, whose functional alteration may lead to compulsive drug use and drug relapse in drug addicts (45). Neuroimaging studies have found increased FC of the orbitofrontal in BQD individuals (46) and also in control subjects immediately after BQ chewing (47). This was in line with our notion that BQ chewing and dependence might affect the brain's reward system. It has also been suggested that the OFC was involved in the processing of rewards and punishments (48), explaining the observed decision-making and goal-driven behavior abnormalities caused by BQD (20). By contrast, a recent fMRI study found that athletes who consumed alcohol or cannabis seemed to have hypoconnectivity in their left OFC relative to nonusers (49), suggesting that different types of psychoactive substances may produce inconsistent neural mechanisms (50).

In our study, the PerAF of the left putamen was decreased. The putamen controls autonomic movements. The putamen injury can interfere with autonomic nervous system functions (51). Furthermore, the putamen within the dorsal striatum influences the acquisition and expression of action-outcome association conditioning (52), which includes the development of habitual compulsive drug dependence. The reduced PerAF of the left putamen in BQD individuals reported here may explain, at least to some degree, the obsessive behavior and drug-seeking in BQD chewers. Similar to other drug dependence users (53, 54), we also found decreased PerAF in BQD individuals in the insula that has been considered a significant structure in generating conscious and interoceptive experiences (55, 56). Similarly, structural imaging studies showed a smaller gray matter volume as insula in addicts (57). Nevertheless, in view of the chicken and egg question, the interpretation of these alterations would still require further investigation.

## Limitations

This study had several limitations. First, we can only observe altered spontaneous brain activity in BQD chewers and are not able to draw direct causal inferences between the BQD and brain activity abnormalities since the study was cross-sectionally designed. Therefore, a longitudinal study design to establish cause-effect relationships should be considered in the future. Second, while the carefully selected samples in this study ensured the comparative specificity of BQ chewing-related problems and limited the use of other addictive substances, alcohol and nicotine consumption are still extremely common in BQD subjects, and their effects could not be fully excluded. Third, the spontaneous brain activity in BQD individuals was evaluated only from single-mode imaging based on the rs-fMRI data. Comprehensive studies on the brain function of BQD using multimodal fusion technology and a more in-depth explanation of genetic mechanisms are lacking. We will study BQD individuals in the future using multimodal fusion technology and genetic imaging to learn more about how gene-environment-brain network-behavior cross-information regulates betel nut addiction at the molecular level.

## Conclusion

We found altered spontaneous neural activity in the impulsivity (areas in the MFG, ACC), cognitive (MFG, ACC, precuneus, and the cerebellum), and reward (orbitofrontal, putamen, and insula) systems in BQD chewers, and this might underline a neurobiological basis for BQD individuals. Moreover, as a new and reliable method, PerAF might be a potential neuroimaging tool to identify spontaneous brain activity alterations in individuals with BQD.

## Data Availability Statement

The datasets in this study are not available currently because the present data is part of an ongoing longitudinal study and most data are still in collection and ought to be protected. Reasonable requests to obtain the data could be emailed to FC, fenger0802@163.com.

## Ethics Statement

This study was formally approved by the Research Ethics Review Committee of the Hainan General Hospital, basing upon the Declaration of Helsinki (2000). The patients/participants provided their written informed consent to participate in this study.

## Author Contributions

All authors listed have made a substantial, direct, and intellectual contribution to the work and approved it for publication.

## Funding

This study was supported by Key Science and Technology Project of Hainan Province (ZDYF2021SHFZ239), the National Nature Science Foundation of China (Grant Nos. 81971602 and 81760308 for FC, Grant No. 81871346 for WH, and Grant No. 82160327 for TL), the Hainan Academician Innovation Platform Fund, and the Hainan Province Clinical Medical Center, Natural Science Research Project of Hainan Medical University (JBGS202107).

## Conflict of Interest

The authors declare that the research was conducted in the absence of any commercial or financial relationships that could be construed as a potential conflict of interest.

## Publisher's Note

All claims expressed in this article are solely those of the authors and do not necessarily represent those of their affiliated organizations, or those of the publisher, the editors and the reviewers. Any product that may be evaluated in this article, or claim that may be made by its manufacturer, is not guaranteed or endorsed by the publisher.
